# Fatal Presumed Re-expansion Pulmonary Edema Following Suction-Assisted Drainage of a Complete Spontaneous Pneumothorax in a Patient With Advanced Emphysema: A Case Report

**DOI:** 10.7759/cureus.111433

**Published:** 2026-06-24

**Authors:** Michal Zielinski, Kamila Dziemianowicz

**Affiliations:** 1 Faculty of Medicine, Uczelni Medycznej im. Marii Skłodowskiej-Curie, Warsaw, POL; 2 Emergency Medicine, Szpital Wolski im. dr Anny Gostyńskiej Sp. z o.o., Warsaw, POL

**Keywords:** chest tube drainage, emergency medicine, emphysema, re-expansion pulmonary edema, respiratory failure, secondary spontaneous pneumothorax

## Abstract

Re-expansion pulmonary edema (RPE) is a rare but potentially life-threatening complication of pleural decompression. Although most reported cases are self-limiting, severe presentations may rapidly progress to respiratory failure and death. We report a fatal case of presumed unilateral RPE following drainage of a complete spontaneous pneumothorax in a patient with advanced emphysema.

A 51-year-old man with chronic obstructive pulmonary disease presented to the emergency department with progressive dyspnea of approximately one week’s duration. On admission, he was ambulatory, hemodynamically stable, and maintained an oxygen saturation of 95% on room air. Computed tomography demonstrated a complete left-sided pneumothorax with near-total collapse of the left lung and advanced bilateral emphysematous changes. A 28 Fr chest tube was inserted and connected immediately to active suction at -20 cm H₂O. Approximately 30 minutes after pleural decompression, the patient developed rapidly progressive hypoxemic respiratory failure. Follow-up chest radiography demonstrated extensive unilateral alveolar-interstitial opacification involving the re-expanded left lung. Despite chest tube revision, endotracheal intubation, mechanical ventilation, and advanced life support, the patient developed recurrent pulseless electrical activity cardiac arrest and subsequently died.

The temporal relationship between pleural drainage and clinical deterioration, together with unilateral radiographic infiltrates and the presence of blood-tinged frothy airway secretions, strongly suggests fulminant RPE as the most likely diagnosis. This case highlights a rare but catastrophic complication of pneumothorax treatment and emphasizes the importance of close monitoring following pleural decompression, particularly in patients with prolonged lung collapse and advanced underlying lung disease.

## Introduction

Re-expansion pulmonary edema (RPE) is a rare but potentially life-threatening complication that may occur following rapid re-expansion of a previously collapsed lung after evacuation of pleural air or fluid [1-4]. Although uncommon, RPE has been reported after treatment of spontaneous pneumothorax, particularly in patients with large or prolonged lung collapse [1,4].

The exact incidence of RPE remains uncertain because many cases are likely underrecognized or clinically mild. Reported incidence varies depending on the studied population, diagnostic criteria, and clinical context. Most cases are mild and self-limited; however, severe presentations resulting in profound hypoxemia, respiratory failure, hemodynamic instability, need for mechanical ventilation, and death have been described [1-4].

The pathophysiology is incompletely understood and is believed to involve a combination of ischemia-reperfusion injury, increased pulmonary capillary permeability, inflammatory mediator release, oxidative stress, and mechanical injury occurring during rapid lung re-expansion [1,2,5].

Several risk factors have been associated with the development of RPE, including prolonged lung collapse, complete pneumothorax, smoking history, underlying lung disease, and the use of negative pleural pressure following chest tube insertion [4,6-8]. These factors are clinically important because they may identify patients who require particularly close monitoring after pleural decompression.

We report a fatal case of presumed unilateral RPE occurring shortly after drainage of a complete spontaneous pneumothorax in a patient with advanced emphysema. This case is presented to highlight the fulminant course that RPE may rarely take and to emphasize the importance of recognizing high-risk clinical features before and after pleural drainage.

## Case presentation

A 51-year-old male smoker with known chronic obstructive pulmonary disease under outpatient pulmonology care presented to the emergency department because of progressive dyspnea. The patient reported a sudden onset of symptoms approximately one week before admission, followed by gradual worsening of respiratory distress. During prehospital management, inhaled salbutamol and intravenous dexamethasone were administered.

On arrival, the patient was conscious, ambulatory, and hemodynamically stable. Initial vital signs were as follows: blood pressure 131/83 mmHg, heart rate 100 beats/min, respiratory rate 25 breaths/min, and oxygen saturation 95% on room air. Physical examination demonstrated markedly diminished breath sounds over the left hemithorax. Point-of-care lung ultrasonography suggested left-sided pneumothorax.

Laboratory investigations demonstrated a C-reactive protein level of 0.09 mg/dL, high-sensitivity troponin I level of 7.7 pg/mL, white blood cell count of 10.3 × 10⁹/L, creatinine level of 0.79 mg/dL, and lactate level of 1.7 mmol/L. Venous blood gas analysis revealed pH 7.336, pCO₂ 51.2 mmHg, and HCO₃⁻ 26.8 mmol/L.

**Figure 1:**
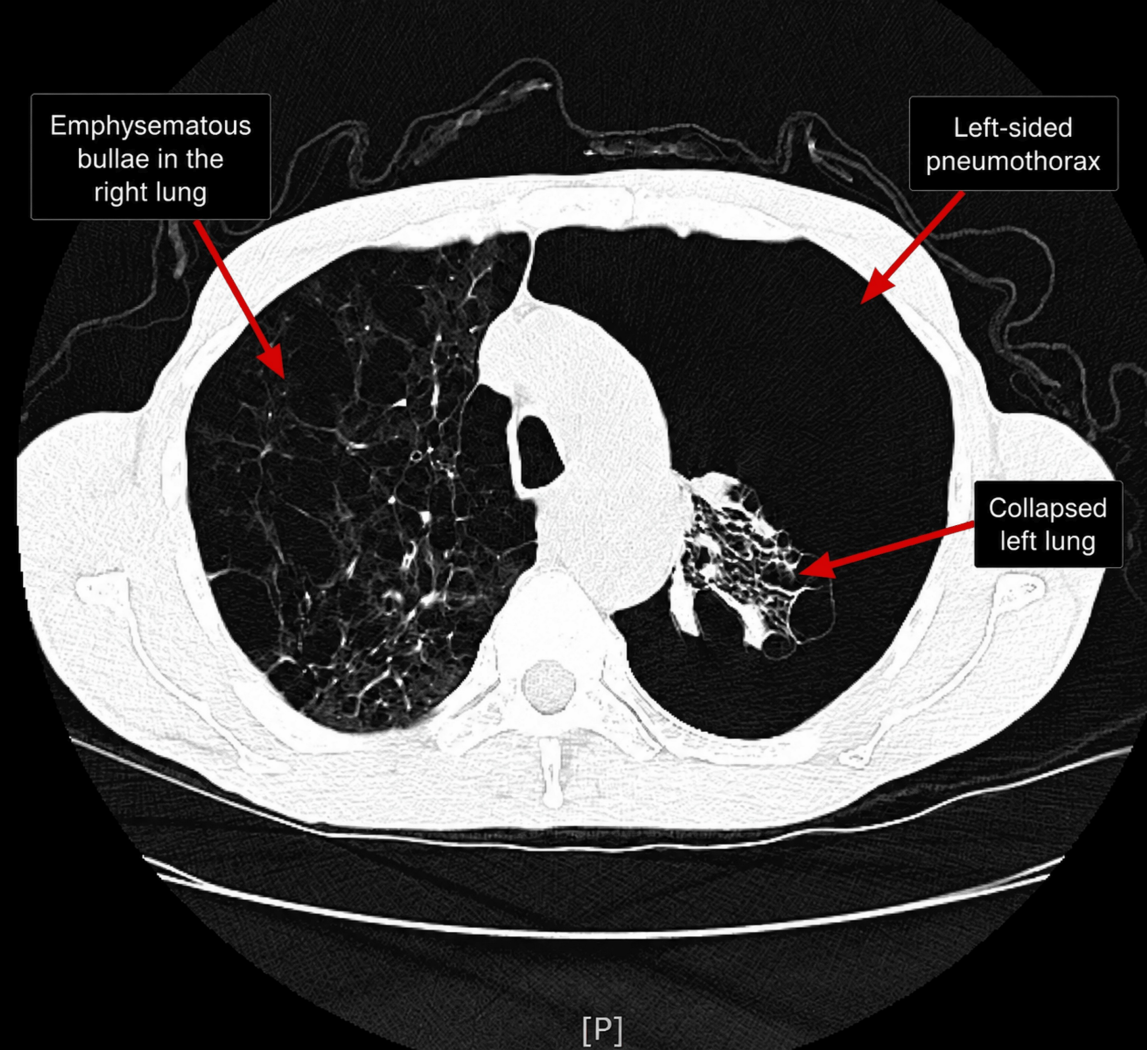
Computed tomography demonstrating a complete left-sided pneumothorax and advanced emphysematous changes Axial computed tomography of the chest obtained before pleural drainage demonstrated a complete left-sided pneumothorax (arrow), with near-total collapse of the left lung (arrow). Advanced emphysematous changes with bullae are visible in the contralateral lung (arrow).

Computed tomography of the chest was performed as the initial imaging study because the patient was hemodynamically stable on presentation. It demonstrated a complete left-sided pneumothorax with near-total collapse of the left lung. Advanced bilateral emphysematous changes and apical bullae were present. No pleural effusion, mediastinal shift, or diffuse pulmonary infiltrates were reported (Figure 1).

**Figure 2:**
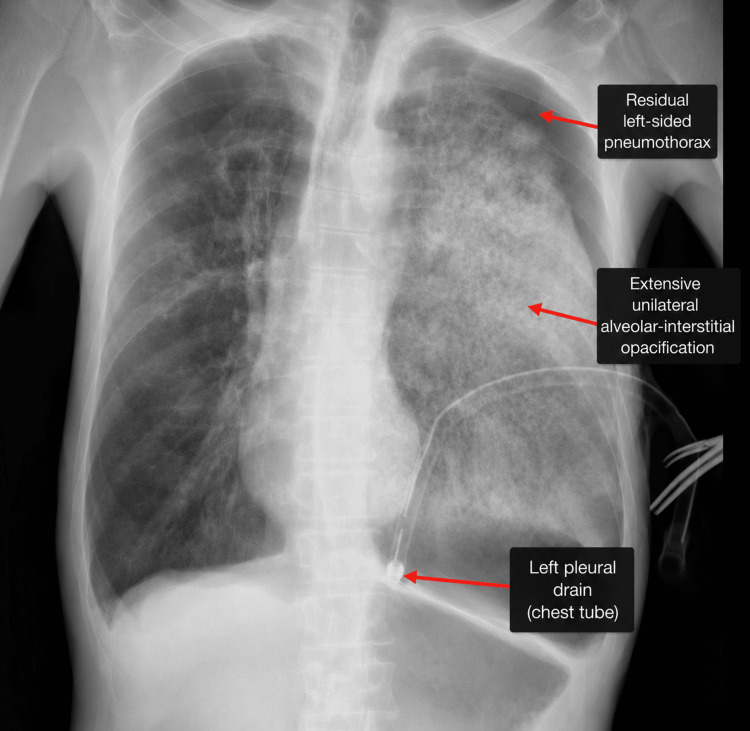
Chest radiograph demonstrating unilateral re-expansion pulmonary edema following pleural drainage Follow-up chest radiograph obtained after pleural drainage demonstrated residual left-sided pneumothorax (arrow); extensive unilateral alveolar-interstitial opacification involving the re-expanded left lung, consistent with presumed re-expansion pulmonary edema (arrow); and a left pleural drain (arrow). The right lung remains relatively spared apart from chronic emphysematous changes.

At approximately 14:30, a 28 Fr chest tube was inserted into the left pleural cavity and connected immediately to active suction at -20 cm H₂O. No initial water-seal drainage period was used. Approximately 30 minutes after drainage, the patient developed rapidly worsening respiratory distress and hypoxemia. A bedside follow-up chest radiograph was then obtained and demonstrated residual left-sided pneumothorax together with extensive unilateral alveolar-interstitial opacification involving almost the entire left lung. The right lung remained relatively spared apart from chronic emphysematous changes (Figure 2).

Thoracic surgical consultation was obtained because of a persistent pneumothorax and concern regarding the effectiveness of drainage. The chest tube was revised; however, no meaningful clinical improvement was observed.

Despite the escalation of oxygen therapy, oxygen saturation remained approximately 80%. Approximately one hour after chest tube insertion, endotracheal intubation was performed by an anesthesiologist. During airway management, abundant blood-tinged frothy secretions were observed within the endotracheal tube. Oxygen saturation transiently improved following intubation and positive-pressure ventilation.

The patient subsequently developed pulseless electrical activity cardiac arrest. Advanced life support was initiated immediately. Return of spontaneous circulation was achieved after approximately 20 minutes. Five minutes later, recurrent cardiac arrest occurred with progression to asystole.

Despite prolonged advanced life support, sustained return of spontaneous circulation could not be achieved. Death was pronounced at 19:00, approximately 7.5 hours after emergency department admission. No postmortem examination was performed.

## Discussion

This case demonstrates a fulminant and ultimately fatal episode of presumed unilateral RPE following drainage of a complete spontaneous pneumothorax.

Several observations support this diagnosis. Computed tomography performed before intervention demonstrated a complete left-sided pneumothorax without diffuse pulmonary infiltrates. Shortly after pleural decompression, extensive unilateral alveolar-interstitial opacities appeared within the previously collapsed lung. Such temporal and radiographic evolution is characteristic of RPE [1-3].

Respiratory deterioration developed rapidly after pleural drainage. The onset of symptoms approximately 30 minutes after lung re-expansion is consistent with previously reported cases of severe RPE [1-4]. Blood-tinged frothy airway secretions were observed during endotracheal intubation. Although not pathognomonic, this finding has been repeatedly described in severe permeability pulmonary edema and supports the proposed diagnosis [1,2,5].

The patient exhibited multiple recognized risk factors for RPE. Symptoms had been present for approximately one week, suggesting prolonged pulmonary collapse. Imaging demonstrated a complete pneumothorax with near-total collapse of the left lung. The patient was an active smoker with advanced emphysematous lung disease, and active suction at -20 cm H₂O was applied immediately after tube thoracostomy. Recent evidence has identified prolonged symptom duration, large pneumothorax size, smoking history, underlying lung disease, and the use of negative pleural pressure as potential risk factors for the development of RPE [4,6-8]. The simultaneous presence of these factors may have increased the patient’s susceptibility to severe re-expansion injury.

Alternative explanations were considered. Infectious pneumonia was considered less likely because inflammatory markers were within the reference range, no infiltrates were identified on pre-drainage computed tomography, and radiographic abnormalities developed within a short interval after pleural decompression. Cardiogenic pulmonary edema was also considered less likely because infiltrates were predominantly unilateral, cardiac biomarkers were not elevated, and clinical deterioration occurred immediately after lung re-expansion. Aspiration-related lung injury cannot be completely excluded; however, the radiographic abnormalities appeared shortly after pleural drainage and before airway instrumentation, making aspiration a less probable explanation.

The exact pathophysiology of RPE remains incompletely understood. Current evidence suggests a multifactorial process involving endothelial injury, increased capillary permeability, inflammatory activation, oxidative stress, and reperfusion injury following rapid re-expansion of a chronically collapsed lung [1,2,5]. These mechanisms may result in disruption of the alveolar-capillary barrier and subsequent accumulation of protein-rich pulmonary edema fluid. Patients with advanced emphysema may be particularly vulnerable because chronic structural destruction of the lung parenchyma and reduced pulmonary reserve may decrease tolerance to rapid re-expansion, mechanical stress, and permeability-related edema.

Recent case reports and case series continue to demonstrate that severe RPE may occur despite apparently appropriate management and may develop even when complete radiographic re-expansion of the lung is not achieved [2,3]. Reported clinical presentations range from mild transient hypoxemia to fulminant respiratory failure requiring mechanical ventilation and intensive care support [2-4,7]. Although most cases are self-limited, severe RPE requiring ventilatory support has been associated with substantial clinical deterioration, and fatal outcomes continue to be reported in the literature [1-3].

Management of RPE remains largely supportive, as no specific therapy has been proven to reverse the underlying pathophysiological process. Early recognition is crucial. Supplemental oxygen should be administered promptly, while patients with severe hypoxemia may require non-invasive ventilation or endotracheal intubation with mechanical ventilation and positive end-expiratory pressure. Although diuretics are frequently used in clinical practice, evidence supporting their routine administration remains limited because increased capillary permeability rather than volume overload appears to be the predominant mechanism of edema formation [1,2,5].

Prevention remains particularly important. Several authors have suggested that patients with prolonged lung collapse, complete pneumothorax, or extensive underlying lung disease may benefit from a more cautious approach to pleural decompression. Avoidance of excessive negative-pressure suction immediately after drainage has been proposed as a strategy to reduce the risk of RPE, although definitive evidence remains limited [1,2,4,6].

In the present case, a large-bore chest tube was connected immediately to active suction at -20 cm H₂O following drainage of a complete pneumothorax with near-total lung collapse. Shortly thereafter, the patient developed rapidly progressive hypoxemic respiratory failure requiring endotracheal intubation and mechanical ventilation. Despite aggressive supportive treatment, respiratory failure progressed to cardiac arrest and ultimately proved fatal. Although a causal relationship between the suction strategy and the development of RPE cannot be definitively established, the clinical course illustrates the potentially fulminant nature of this complication and underscores the importance of close monitoring after pleural decompression in high-risk patients.

This report has several limitations. No postmortem examination was performed, preventing definitive pathological confirmation of the diagnosis. Arterial blood gas analysis following deterioration, transthoracic echocardiography, and repeat computed tomography after development of pulmonary infiltrates were unavailable because of the rapidly progressive clinical course. Consequently, the diagnosis remains presumptive and is based on the temporal relationship to pleural drainage together with characteristic clinical and radiographic findings.

## Conclusions

RPE is a rare but potentially fatal complication of pneumothorax drainage. Clinicians should maintain a high index of suspicion when severe hypoxemia and unilateral pulmonary infiltrates develop shortly after pleural decompression, particularly in patients with prolonged lung collapse, complete pneumothorax, smoking history, or advanced underlying lung disease.

This case demonstrates how presumed unilateral RPE may progress rapidly from initial respiratory deterioration to refractory cardiac arrest despite aggressive supportive treatment. Careful monitoring after pleural decompression and awareness of this uncommon complication remain essential, especially in patients considered to be at increased risk.
